# Navigating Diagnostic Delays: A Case of Takayasu Arteritis Complicated by Acute Heart Failure and Vascular Compromise in a 37-Year-Old Woman From Cameroon

**DOI:** 10.7759/cureus.83058

**Published:** 2025-04-27

**Authors:** Tintinu Edmond Asongtia, Tchinda Gerald Fomekong, Lambi Jervis Somo, Feudjio Saapi Ghislain, Keba Faith Bih Irene

**Affiliations:** 1 Internal Medicine, Mbingo Baptist Hospital, Bamenda, CMR; 2 Radiology, Mbingo Baptist Hospital, Bamenda, CMR; 3 Surgery, Mbingo Baptist Hospital, Bamenda, CMR

**Keywords:** blood pressure discrepancy, congestive heart failure, late diagnosis, secondary hypertension, takayasu arteritis

## Abstract

Takayasu arteritis (TAK) is a rare form of large-vessel vasculitis that significantly impacts cardiovascular health, leading to severe complications such as acute heart failure (AHF). Its rarity in Cameroon may be attributed to limited awareness, underdiagnosis, and a lack of published data on the condition. We report the case of a 37-year-old female patient who presented with AHF and secondary hypertension, chest pain, and AHF after undergoing umbilical hernia repair two weeks prior. Initial symptoms included dyspnea at rest (New York Heart Association (NYHA) stage 4), orthopnea, paroxysmal nocturnal dyspnea, body swelling, and chest pain. Her medical records indicated mildly elevated blood pressure and an inter-arm blood pressure discrepancy, which previous healthcare providers had overlooked. On examination, she exhibited signs of respiratory distress, jugular venous distention, and a notable discrepancy in blood pressure between her arms. Investigations revealed elevated inflammatory markers (CRP: >200 mg/L), and significant findings on imaging, notably, a point-of-care bedside cardiac ultrasound showed a left ventricular ejection fraction of 30%. An arterial duplex ultrasound demonstrated complete occlusion of the left common carotid artery and severe stenosis of the left subclavian artery, supporting a diagnosis of AHF complicating TAK during the pulseless phase. The patient also met four of the minor Ishikawa criteria, including elevated erythrocyte sedimentation rate (ESR) and significant aortic insufficiency. Treatment was promptly initiated with furosemide for AHF management, followed by lisinopril and bisoprolol as her hypervolemic state improved. High-dose corticosteroids were prescribed to address the vasculitis. The patient showed clinical improvement, with dyspnea at rest resolving and her functional status improving to NYHA stage 3. However, she later developed renal impairment attributed to cardiorenal syndrome and requested transfer to a closer medical facility. While plans for further management and control of inflammatory markers were made, she unfortunately passed away a few weeks after discharge. This case underscores the challenges in diagnosing and managing TAK, especially in regions where the disease is rare, highlighting the need for heightened clinical suspicion in similar presentations.

## Introduction

Takayasu arteritis (TAK) is a chronic inflammatory disease of unknown etiology, characterized by granulomatous inflammation of medium and large arteries, primarily affecting the aorta and its branches. The disease predominantly affects women (80-90% of cases) and typically presents between the ages of 10 and 40 years [1]. Although TAK is found worldwide, it has the highest prevalence in Asia [2]. In Africa, most reported cases originate from Tunisia [3], with only one documented case in Cameroon [4].

TAK poses a significant global health burden due to its potential for severe vascular complications, including heart failure, stroke, and organ ischemia. Its rarity and atypical presentations often lead to underdiagnosis, particularly in low-resource settings. In Africa, factors such as limited access to healthcare infrastructure, advanced diagnostic imaging, and specialized training contribute to a lack of awareness among healthcare providers, resulting in delayed diagnosis and treatment [5]. This gap in recognition is crucial, as early intervention can significantly improve patient outcomes.

TAK exhibits a triphasic pattern of expression, comprising a systemic nonvascular phase, a vascular inflammatory phase, and a quiescent “burnt-out” phase [6]. During the systemic nonvascular phase, patients may experience nonspecific symptoms such as fever and fatigue. The vascular inflammatory phase is marked by significant arterial involvement, leading to symptoms like claudication and hypertension. The quiescent "burnt-out" phase occurs when symptoms may diminish, but irreversible vascular damage has already taken place, resulting in long-term cardiac or renal complications.

Patients with TAK who receive early and effective intervention tend to have a more favorable prognosis. Studies indicate that aggressive management can help mitigate severe vascular complications, such as heart failure, stroke, and ischemia, significantly improving survival rates. However, even with treatment, there is a notable risk of recurrent complications. Ongoing management of TAK typically involves a multidisciplinary approach focusing on immunosuppressive therapies, symptomatic treatment, and regular monitoring. Immunosuppressive medications, such as corticosteroids alongside agents like methotrexate, play a crucial role in controlling inflammation and preventing relapse. Patients require regular imaging and clinical evaluations to monitor disease activity and assess any vascular morbidity [7]. 

This report highlights a young Cameroonian female patient who developed acute heart failure (AHF), presenting in the late vascular phase of TAK, characterized by established vascular damage and critical complications. Late diagnosis is particularly problematic because the progressive nature of TAK can lead to severe organ ischemia and damage, significantly impairing patient outcomes. AHF can occur in TAK due to several mechanisms, including the development of significant aortic and mitral regurgitation caused by vascular inflammation and structural changes, resulting in volume overload on the heart. Additionally, chronic hypertension, a common consequence of arterial involvement, can lead to left ventricular hypertrophy and eventually heart failure [8]. This case underscores the importance of thorough physical examinations and modern imaging techniques in diagnosing TAK, particularly in atypical presentations.

## Case presentation

The patient was a 37-year-old woman who had undergone an umbilical hernia repair two weeks before the hospital visit. She presented with worsening chest pain and AHF symptoms, including dyspnea at rest (New York Heart Association (NYHA) stage 4), orthopnea, paroxysmal nocturnal dyspnea, and body swelling. The chest pain was central, nonradiating, and associated with retrosternal pressure. These symptoms had lasted two weeks. Fever, myalgias, or limb claudication were absent. She had a history of chronic dyspepsia, for which a gastroscopy had suggested erosive antral gastritis. Her records over the preceding year during evaluation for dyspepsia showed an elevation in blood pressure with average systolic and diastolic blood pressure ranges of 140-150 mmHg and 90-100 mmHg, respectively, all measured on the right arm. Additionally, C-reactive protein (CRP) was elevated (>200 mg/L; normal range <5 mg/L) but went unheeded. She had never received treatment for hypertension. The only medication prescribed was intermittent omeprazole. Otherwise, her cardiovascular history was uneventful. Her family and social histories were unremarkable.

On presentation, the patient was acutely ill and was in respiratory distress. Vital signs, including respiratory rate (22 breaths/min), oxygen saturation (95%) on room air, and heart rate (109 bpm), were recorded. Brachial blood pressure recording revealed a significant discrepancy in the blood pressure of the upper limbs: 149/63 mmHg in the right arm and 104/59 mmHg in the left arm. 

Her physical examination revealed jugular venous distention up to the angle of the jaw and a heart’s apex beat displaced to the 6th intercostal space at the left anterior axillary line, with an apical pansystolic murmur and an early diastolic murmur over the left lower sternal edge. Fine crackles were heard bilaterally in the lower thoracic region, and she had bilateral pedal edema. The left carotid and left radial pulses were weak; pulses were normal everywhere else. Tenderness was observed over the left carotid artery, and a bruit was heard on auscultation. There was no abdominal bruit. Other systemic examination findings were unremarkable. Differential diagnoses included vasculitis, myocarditis, acute coronary syndrome (ACS), aortic dissection, and valvular heart disease complicated by AHF.

**Figure 1:**
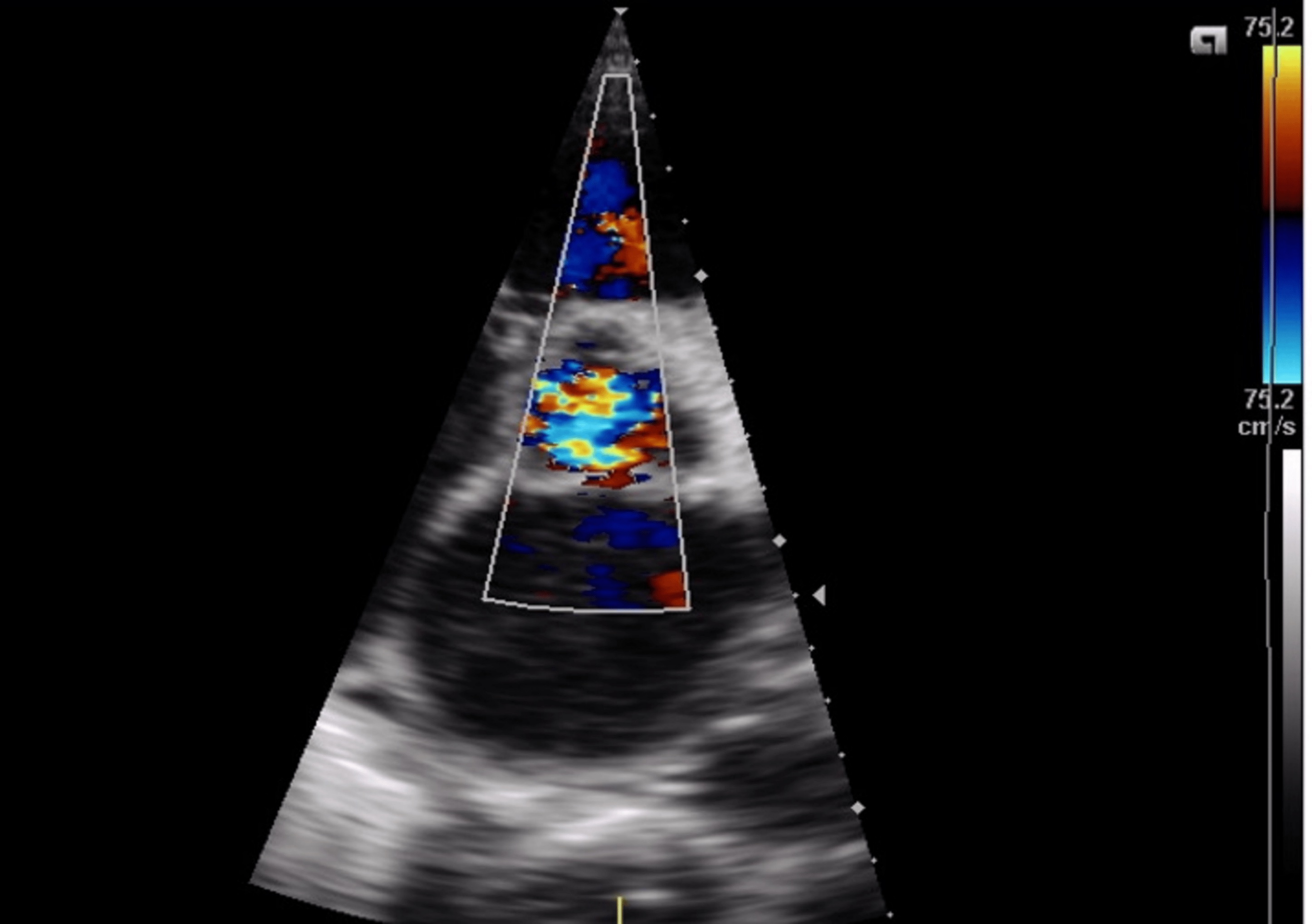
: Parasternal short-axis view of the aortic valve with color Doppler Imaging showing severe aortic insufficiency

**Figure 2:**
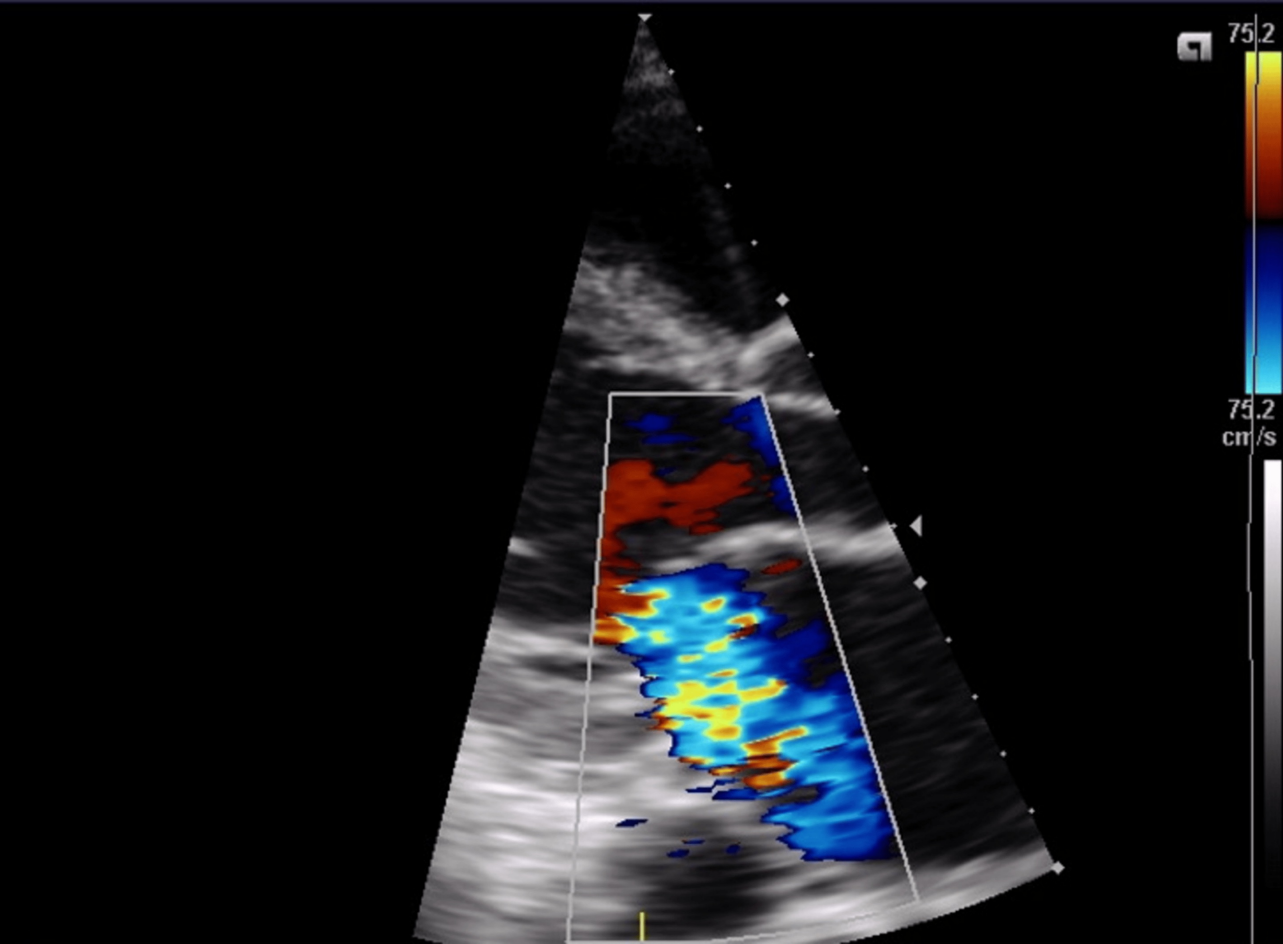
Parasternal long-axis image of the heart with color Doppler Imaging showing severe mitral regurgitation

**Figure 3:**
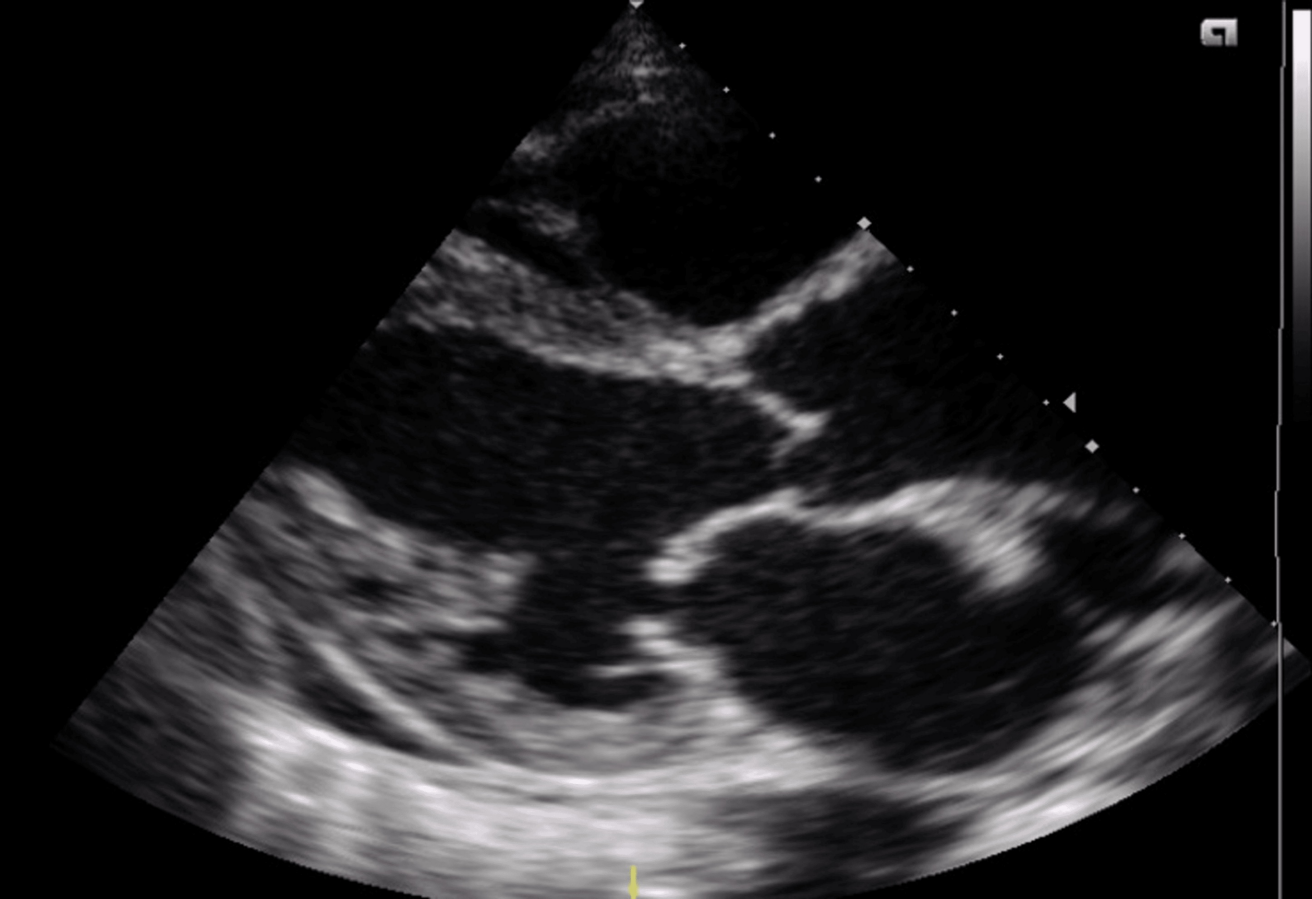
Parasternal long axis of the heart showing dilated chambers

**Figure 4:**
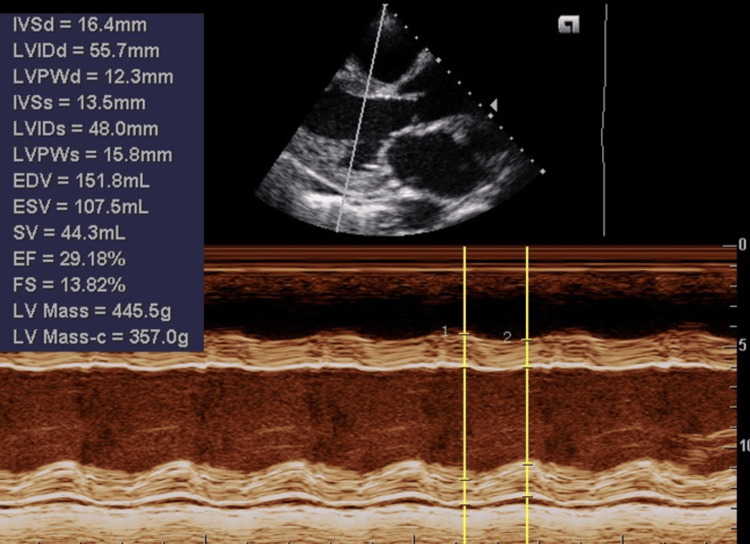
M-mode tracing of the left ventricle with measurements giving an ejection fraction of 29%, dilated left ventricle, increased end-diastolic volume (EDV) = 151 ml, and end-systolic volume (ESV) = 107 ml

Electrocardiography (ECG) showed sinus tachycardia, left axis deviation, and left ventricular hypertrophy with left atrial enlargement. Chest radiography revealed congested lung fields with bilateral blurred costophrenic angles and cardiomegaly. Echocardiography revealed a reduced left ventricular ejection fraction of 30% and severe aortic and mitral regurgitation with a severely dilated left atrium and ventricle (Figures 1-4). Her blood investigations, including full blood count and renal and liver function tests, were normal. Troponin I was not measured.

**Figure 5:**
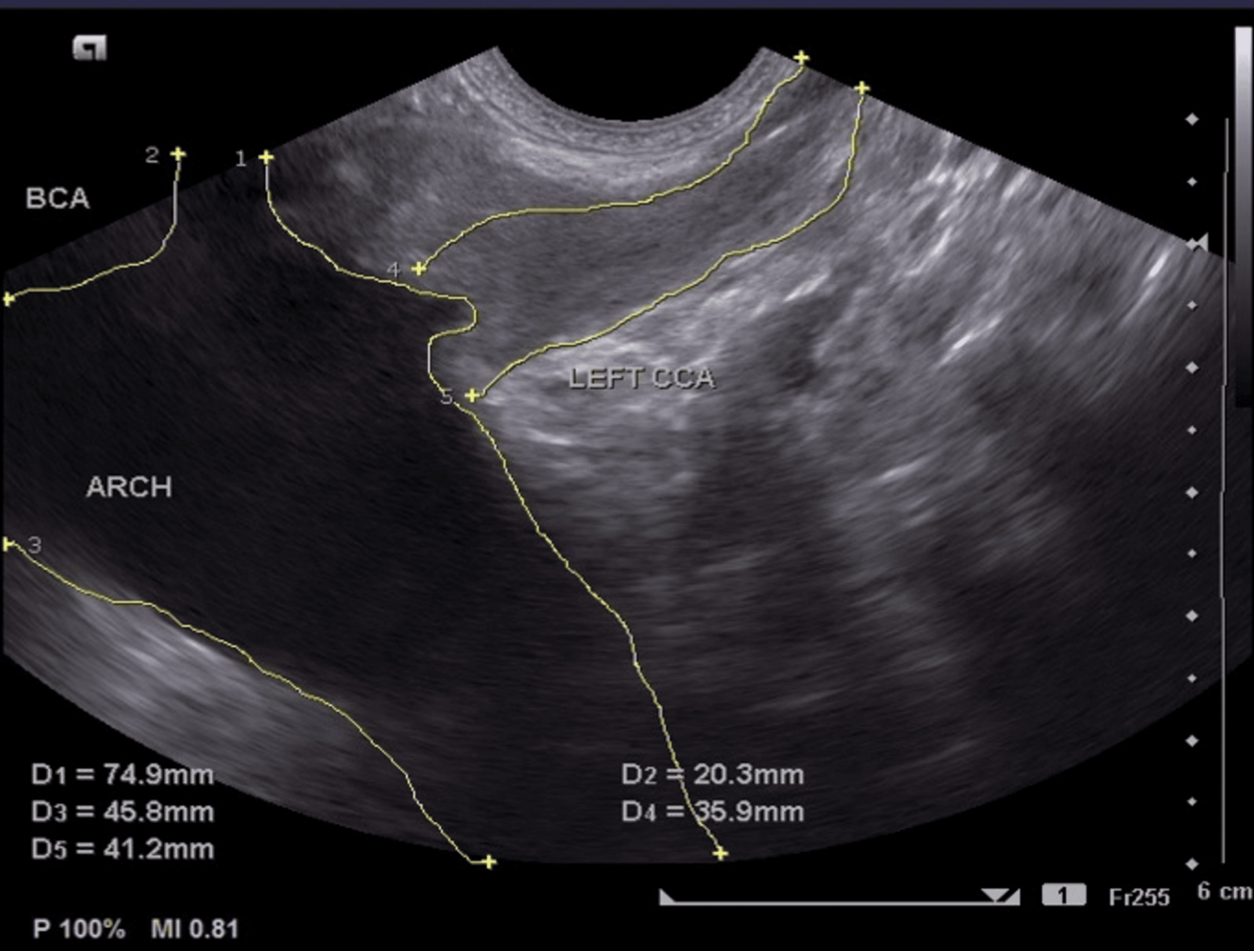
2D sonogram of the aortic arch depicting complete occlusion of the entire left CCA (blue arrow) 2D: two dimensional; BCA: brachiocephalic artery; CCA: common carotid artery

**Figure 6:**
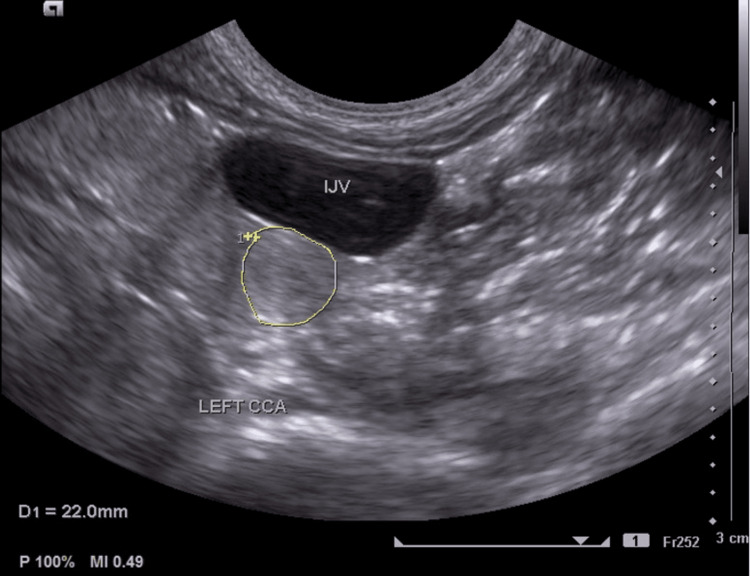
Short-axis view of the left neck showing the internal jugular vein and occluded left CCA IJV: Internal jugular vein; CCA: common carotid artery

**Figure 7:**
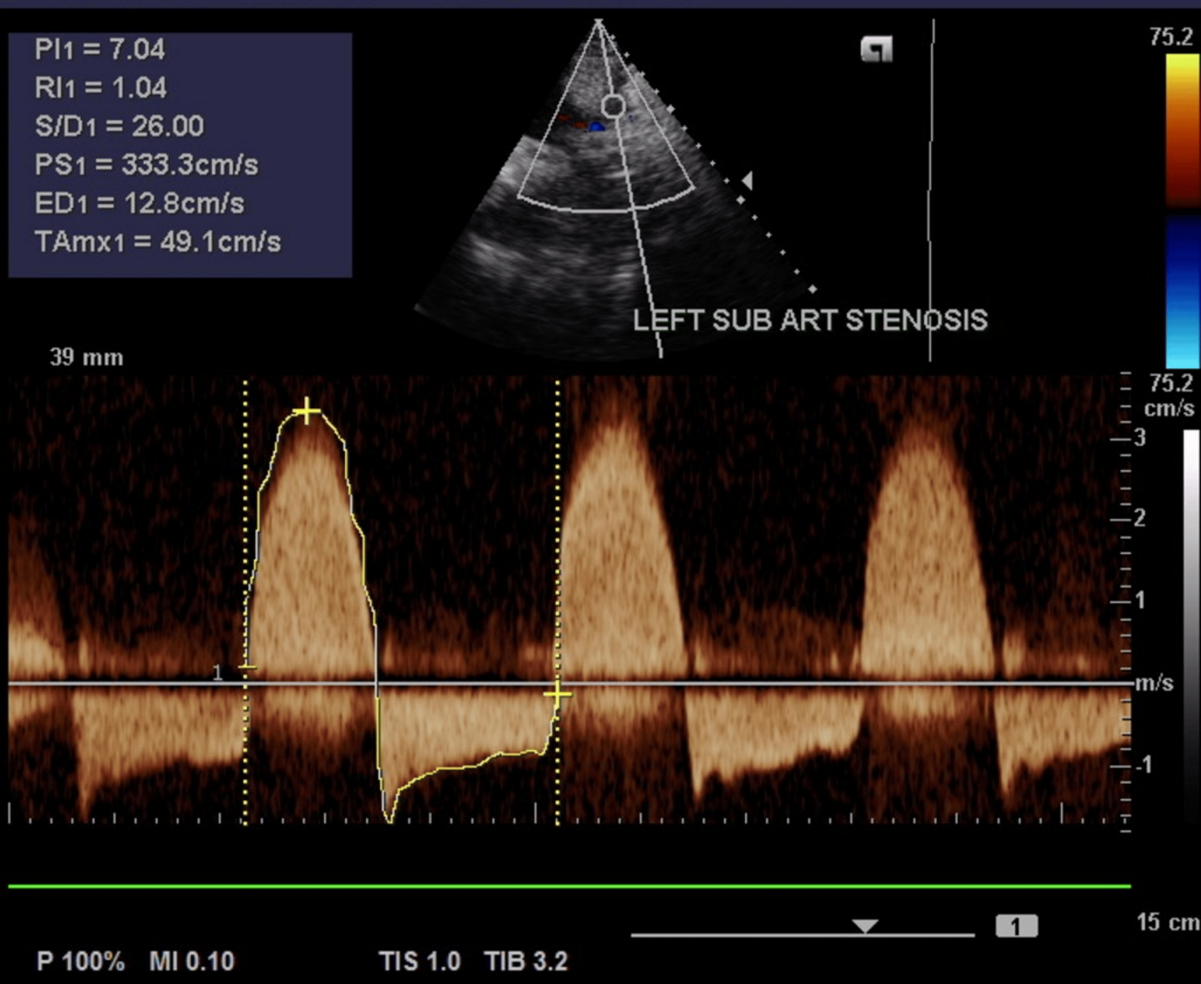
Spectral Doppler (continuous wave Doppler) of the proximal segment of the left subclavian artery. Note the high peak velocities of 333 cm/s (normal range 45-180 cm/s) P: positivity; R: resistivity; S/D: systolic/diastolic; PS: peak velocity; ED: end diastolic velocity; TAmax: mean volocity

CRP (172.82 mg/l; normal range <5 mg/L) and erythrocyte sedimentation rate (ESR) (70 mm/h; normal range: 0-20 mm/h) were elevated. Gene Xpert on gastric aspirate, syphilis serology, and human immunodeficiency virus serology were negative. An arterial duplex ultrasound of the neck vessels showed complete occlusion of the left common carotid artery, external carotid artery, and internal carotid artery. There was severe stenosis of the proximal segment of the left subclavian artery proximal to the origin of the left vertebral artery. Abnormal flow in the left vertebral artery was consistent with the incomplete subclavian steal phenomenon (Figures 5-7).

The working diagnosis was AHF complicating TAK. The differential diagnoses included myocarditis, ACS, aortic dissection, and valvular heart disease. TAK in the vascular phase was considered based on the high-grade stenosis of the proximal portion of the left subclavian and left carotid arteries. Additionally, the patient fulfilled four of the minor Ishikawa Criteria (Table 1) [9], including an ESR of >20 mm/h, sensitivity of the left carotid artery to palpation, elevated blood pressure, and aortic insufficiency.

**Table 1:** Modified Ishikawa Criteria for the diagnosis of Takayasu arteritis This table was adapted/reproduced from Sharma et al. [9], with permission

Major criteria	Minor criteria
Stenosis or occlusion of the middle portion of the left subclavian artery on the arteriography	1. ESR >20 mm/h
Stenosis or occlusion of the middle portion of the right subclavian artery on the arteriography	2. Sensitivity of carotid arteries to palpation
Characteristic symptoms lasting at least one month: claudication, absence of a pulse or blood pressure asymmetry, fever, cervicalgia, amaurosis, visual disorders, syncope, dyspnea, and palpitations	3. High blood pressure: humeral pressure-140/90 mmHg, or popliteal pressure-160/90 mmHg
	Aortic insufficiency or annuloaortic ectasia
	Pulmonary arterial impairment
	Stenosis or occlusion of the middle portion of the left carotid artery on the arteriography
	Stenosis or occlusion of the distal third of the brachiocephalic artery trunk on the arteriography
	Impairment of the descending thoracic aorta on the arteriography
	Impairment of the abdominal aorta on the arteriography
	Coronary heart injury before the age of 30, in the absence of dyslipidemia or diabetes
A diagnosis of Takayasu’s arteritis is highly likely if: ≥2 major criteria or 1 major criterion and ≥2 minor criteria or ≥4 minor criteria	

ACS was strongly considered given the nature and location of the chest pain, specifically, pressure-like and central. Unfortunately, serum troponin levels were unavailable at the time of assessment. However, the negative electrocardiogram (EKG) for ischemia and the cardiac ultrasound, which did not show regional wall motion abnormalities, coupled with a rapid improvement in the patient’s symptoms following initial management with diuretics and corticosteroids, made ACS less likely. Aortic dissection/coarctation was ruled out based on a negative chest CT angiogram.

Myocarditis was considered due to the AHF symptoms associated with chest pain. However, the patient denied experiencing a preceding upper respiratory infection, and myocarditis would not account for the observed pulse deficits and significant blood pressure discrepancies between both arms.

The patient was promptly initiated on furosemide in addition to fluid and salt restriction for AHF. Lisinopril and bisoprolol were subsequently added when the hypervolemic state improved. Vasculitis was addressed with high-dose corticosteroids (prednisone 60 mg/day). Her hospital course was marked by improved dyspnea. The dyspnea at rest resolved; dyspnea was only noticed after walking on a flat surface for 100 meters. This resulted in an improvement in her functional status to NYHA stage 3.

Subsequently, she developed renal impairment, which was attributed to cardiorenal syndrome. Due to financial constraints, she requested to be transferred to a different medical facility closer to her home. Control of inflammatory markers and the addition of methotrexate were envisaged after the active phase of her illness. However, the patient died a few weeks following discharge from our hospital. It remains unclear whether this was due to progression of heart failure, renal failure, or another complication such as sudden cardiac death, infection, or noncompliance with treatment. This uncertainty underscores the need for careful follow-up and further investigation into the causes of postdischarge mortality in similar cases. The following table summarizes the Modified Ishikawa Criteria utilized for diagnosing TAK. These criteria combine clinical symptoms, imaging findings, and laboratory results.

## Discussion

This case highlights several critical issues in the diagnosis and management of TAK, particularly within the context of healthcare in Sub-Saharan Africa and Cameroon. The complexities surrounding the patient's presentation, diagnostic lapses, and management decisions reveal systemic challenges that can delay appropriate care.

In this case, there was a vague temporal link between the umbilical hernia repair and the onset of the patient’s symptoms. While it is plausible that the surgery may have precipitated her condition, particularly through changes in hemodynamics or stress [10], it could also be coincidental.

ACS was strongly considered given the nature and location of the chest pain, specifically, pressure-like and central. Unfortunately, serum troponin levels were unavailable at the time of assessment. However, the negative EKG for ischemia and the cardiac ultrasound, which did not show regional wall motion abnormalities, coupled with a rapid improvement in the patient’s symptoms following initial management with diuretics and corticosteroids, made ACS unlikely. The pain was attributed to her underlying vasculitis [11]. Aortic dissection was ruled out by a negative chest CT angiogram.

Myocarditis was considered due to the AHF symptoms associated with chest pain. However, the patient denied experiencing a preceding upper respiratory infection, and myocarditis would not account for the observed pulse deficits and significant blood pressure discrepancies between both arms. 

The elevated blood pressure and raised CRP levels noted one year prior to her presentation are significant indicators of underlying pathology that may have been overlooked. This situation reflects a missed opportunity for earlier intervention and emphasizes a potential system-level gap in patient follow-up and awareness regarding the implications of chronic hypertension and elevated inflammatory markers in a young female patient.

The patient’s delayed diagnosis of TAK exemplifies a broader issue in healthcare systems, particularly in low-resource settings. A contributing factor to the diagnostic delay was the low awareness of vasculitis among primary care providers. Many healthcare professionals in these regions may not be sufficiently familiar with the varied manifestations of vasculitis, leading to oversights in diagnosis. A study by Dahl et al. [12] noted that increased awareness and education on rare diseases among general practitioners could markedly improve early diagnosis and management.

Additionally, there are very few published cases of TAK in Sub-Saharan Africa, particularly in Cameroon. The scarcity of literature can hinder healthcare providers' ability to recognize the condition promptly, making it more difficult to apply appropriate clinical guidelines or consider rare diagnoses effectively. Limited case reports and clinical guidelines in Africa reinforce the disparities in disease awareness and management.

The decision to perform carotid and cardiac Doppler ultrasounds prior to a chest angiogram was clinically justified, given the patient's specific symptoms and the need for immediate information regarding her vascular status. Doppler ultrasounds are noninvasive and can quickly assess blood flow and structural abnormalities in the arteries, particularly focusing on the carotid circulation, driving cerebral perfusion. The results indicated complete occlusion of the left common carotid artery and subclavian stenosis, which were vital in establishing a working diagnosis of TAK.

While a chest angiogram could have provided valuable information about the aorta and its branches, it may have been less urgent in guiding initial management compared to the immediate need to assess symptomatic vascular territories affecting cerebral and cardiac function. Furthermore, the emergence of AHF symptoms necessitated prioritizing interventions that could improve hemodynamics swiftly. As reported by James et al. [13], early imaging can lead to quicker therapeutic decisions, which is significant in acute settings.

Upon further evaluation with angiography, additional information could have potentially illuminated the extent of systemic involvement and aortic abnormalities. Such insights would be instrumental for long-term management strategies, particularly if surgical interventions were considered. Park et al. [14] identified that timely angiographic evaluation can often reveal critical lesions that alter the trajectory of patient care.

In this case, initiating treatment with high-dose corticosteroids as monotherapy was appropriate given the acute nature of the patient's symptoms and the need for rapid anti-inflammatory effects. While immunosuppressants like methotrexate or azathioprine are often used in the chronic management of TAK to mitigate long-term immunosuppression, they require more time to achieve clinical benefit and carry their own risk of complications, especially if the patient is in a hypervolemic state or has renal impairment. Starting with steroids allows for quicker symptom relief and stabilization, as emphasized by Gonzalez-Gay et al. [15].

The patient's eventual demise raises critical questions regarding the causative factors involved. Progressively worsening renal failure, potentially exacerbated by aggressive diuretic therapy and underlying cardiorenal syndrome, might have played a significant role. Sudden cardiac death could also be plausible, given the patient's severely reduced ejection fraction and history of myocardial strain. Nonadherence to treatment postdischarge, compounded by financial constraints, may have adversely affected her prognosis. The lack of comprehensive follow-up care after discharge likely contributed to her adverse outcome, as discussed by Kishore et al. [16], who noted that continuity of care significantly impacts outcomes for patients with chronic diseases.

Diagnosis and management

Diagnosing and managing rare diseases like TAK poses inherent challenges, particularly in low-resource settings. Insufficient medical training and the absence of established diagnostic protocols challenge healthcare providers’ abilities to recognize and treat these conditions effectively. Additionally, with limited access to advanced imaging and diagnostics, healthcare providers may resort to treating symptoms rather than underlying causes, as seen in this case. To mitigate misdiagnosis and improve management of TAK in similar settings, several recommendations can be proposed:

Blood Pressure Screening in Both Arms

Routine measurement of blood pressure in both arms could help identify discrepancies indicative of vascular pathologies such as TAK.

Educational Initiatives

Implementing continuing medical education programs focused on rare diseases, including TAK, would enhance clinician awareness and recognition.

Strengthen Referral Pathways

Establishing clear pathways for referral to specialized centers for suspected cases of vasculitis could significantly promote timely diagnosis and treatment.

Enhanced Access to Imaging

Expanding access to advanced imaging modalities, both Doppler and angiographic studies, should be prioritized within resource-constrained healthcare settings to support timely diagnosis.

Furthermore, the application of the Modified Ishikawa Criteria in this case provided a structured, systematic approach to diagnosis. By integrating these criteria, which emphasize identifying contributing factors and underlying issues in the diagnostic process, healthcare providers can develop a clearer understanding of complex cases like TAK. The utility of these criteria reinforces the importance of a comprehensive framework in recognizing rare diseases, fostering timely intervention, and ultimately improving patient outcomes. Adopting such methodologies could significantly enhance diagnostic accuracy and care delivery in low-resource environments.

## Conclusions

The diagnosis of TAK must be considered in young adults presenting with secondary hypertension, particularly when associated with inter-arm systolic blood pressure discrepancy. Other findings that should increase our index of suspicion for TAK include limb claudication in a younger individual, pulse deficits, and an unexplained increase in acute-phase reactants. Late diagnosis of TAK is associated with poor outcomes.
